# A comparison of physical activity and sedentary behaviour in 9–11 year old British Pakistani and White British girls: a mixed methods study

**DOI:** 10.1186/1479-5868-11-74

**Published:** 2014-06-09

**Authors:** Yvonne C Hornby-Turner, Kate R Hampshire, Tessa M Pollard

**Affiliations:** 1 Physical Activity Lab, Department of Anthropology, Durham University, Dawson Building, Stockton Road, Durham DH1 3LE, UK

**Keywords:** Ethnicity, Accelerometry, Qualitative, South Asian, European, Screen-time, Active travel, Sport, Outdoor play

## Abstract

**Background:**

Previous studies suggest that British children of South Asian origin are less active and more sedentary than White British children. However, little is known about the behaviours underlying low activity levels, nor the familial contexts of active and sedentary behaviours in these groups. Our aim was to test hypotheses about differences between British Pakistani and White British girls using accelerometry and self-reports of key active and sedentary behaviours, and to obtain an understanding of factors affecting these behaviours using parental interviews.

**Methods:**

Participants were 145 girls (70 White British and 75 British Pakistani) aged 9–11 years and parents of 19 of the girls. Accelerometry data were collected over 4 days and girls provided 24-hour physical activity interviews on 3 of these days. Multilevel linear regression models and generalised linear mixed models tested for ethnic differences in activity, sedentary time, and behaviours. Semi-structured interviews were conducted with parents.

**Results:**

Compared to White British girls, British Pakistani girls accumulated 102 (95% CI 59, 145) fewer counts per minute and 14 minutes (95% CI 8, 20) less time in moderate to vigorous physical activity per day. British Pakistani girls spent more time (28 minutes per day, 95% CI 14, 42) sedentary. Fewer British Pakistani than White British girls reported participation in organised sports and exercise (OR 0.22 95% CI 0.08, 0.64) or in outdoor play (OR 0.42 95% CI 0.20, 0.91). Fewer British Pakistani girls travelled actively to school (OR 0.26 95% CI 0.10, 0.71). There was no significant difference in reported screen time (OR 0.88 95% CI 0.45, 1.73). Parental interviews suggested that structural constraints (e.g. busy family schedules) and parental concerns about safety were important influences on activity levels.

**Conclusions:**

British Pakistani girls were less active than White British girls and were less likely to participate in key active behaviours. Sedentary time was higher in British Pakistani girls but reported screen-time did not differ, suggesting that British Pakistani girls engaged more than White British girls in other sedentary behaviours. Interviews highlighted some differences between the groups in structural constraints on activity, as well as many shared constraints.

## Introduction

Adults of South Asian origin living in the UK, Canada and the US have a higher risk of type 2 diabetes and cardiovascular disease than those of European origin [1-3] and differences between these groups in risk factors for these diseases have also been observed in young British children [4]. It is possible that differences in levels of physical activity and sedentary time are partly responsible for differences in risk factor levels in childhood; low levels of physical activity are associated with markers of disease risk in children [5] and objectively assessed sedentary time has been found to be associated with health risk in children in some studies (e.g. [6] (but not in others e.g. [7]). Questionnaire studies suggested that British South Asian children are less active than White British children [8], and lower activity in British South Asian compared to White British children has been confirmed using accelerometry [9,10] and pedometry [11]. South Asian children also recorded more minutes of sedentary time than White British children [9,10].

Much less is known about the differences in behaviour that underlie these differences in activity levels. In children, more participation in organised sports or exercise, in outdoor play and in active travel to school have been shown to contribute to objectively assessed total physical activity and to the percentage of time spent in moderate to vigorous activity (MVPA) [12-14]. Descriptive data from the Health Survey for England based on recall for the past seven days suggest that children of South Asian origin (with the exception of boys of Indian origin) may be less likely to participate in sports and exercise, or in active play, than children from the general population [15]. A lower proportion of girls of Pakistani origin, but not of boys of Pakistani origin or girls or boys of other South Asian ethnicities, were reported to walk for 5 minutes or more per week compared to the general population. Owen et al. [16] found that South Asian children were less likely than White European children to walk or cycle to school. More information on behaviours underlying low levels of activity in children of South Asian origin is required.

Screen-time is the most commonly researched sedentary behaviour and it appears to be more strongly associated with risk of obesity and cardiometabolic disease than is objectively assessed sedentary time [17]. While screen-time has been shown to correlate with objectively assessed sedentary time in children, the association does not appear to be strong [18]. Two studies have compared self-reported screen-time in British South Asian and White British children and found no difference [19,20].

As in other ethnic groups, girls of South Asian origin are less active than boys [9]. Although many studies consider those of South Asian origin as one group, there appears to be heterogeneity in activity behaviours by country of origin, with those of Pakistani and Bangladeshi origin generally reporting lower levels of activity than those of Indian origin [8].

We therefore conducted a focused and detailed mixed methods study of activity and sedentary time comparing British Pakistani girls, who might be expected to be a particularly inactive South Asian group, with White British girls. We set out to compare objective measures of activity, to characterise differences between this group and White British girls in key active and sedentary behaviours using optimised methods of self-report, and to interview parents in an effort to understand the family context of these behaviours. We tested predictions that British Pakistani girls would be less active and more sedentary, as assessed by accelerometer, than White British girls and that British Pakistani girls would engage less in active behaviours (organised sports and exercise, outdoor play, active travel to school) but expected no difference in screen-time compared with White British girls. We considered it important to provide a more complete picture of activity levels and behaviours underlying activity as it is only when behaviours and their contexts are understood that effective intervention studies can be designed.

## Methods

### Study design and participants

In order to recruit year 5 (aged 9–10 years) and year 6 (aged 10–11 years) girls to the study, 8 primary schools in a large economically deprived conurbation in North East England known to have a relatively high proportion of British Pakistani children were approached.

Parents of participating girls were sent a letter asking if they would be willing to participate in an interview. Informed written parental consent and verbal child assent to participate was provided by all participants. The research design and protocol received ethical approval from Durham University ethics committee. Data were collected between June 2009 and December 2010. No data were collected during Ramadan (21 August - 19 September 2009 and 11 August – 9 September 2010).

### Measures

#### Questionnaire

Parents were asked to complete a general socio-demographic questionnaire. Ethnicity was based on parentally defined ethnicity for parents if provided, otherwise geographic origin of parents and grandparents, otherwise parentally defined ethnicity of child, otherwise child-defined geographic origin of parents and grandparents.

#### Accelerometry

All girls were asked to wear the ActiGraph GT3X (ActiGraph, FL, USA) accelerometer on their left hip from Friday morning to Wednesday morning during the school term. The Actigraph accelerometer is validated for measuring physical activity in children of this age [21]. On Friday morning girls were shown how to wear the monitor on a belt around the waist and were asked to wear it during waking hours, except when bathing, showering or swimming. Data were recorded in 5 second epochs. The data were processed using a dedicated software programme (MAHUFFE, available from http://www.mrc-epid.cam.ac.uk/research/resources/materials-transfer-disclaimer/physical-activity-downloads). Periods of twenty minutes or more of continuous zero activity counts, which were considered to indicate non-wear, were removed [9]. Data from the Friday were discarded and data collected outside the hours of 7 am and 11 pm were excluded from analysis [22]. Only participants with at least 3 days, including 2 weekdays, of at least 500 minutes of data each (a lower value than is typical for adults to take account of the lower number of wakeful hours in children), were included in analyses [21,23]. We used the same cut-points as Owen et al. [9] in their study of ethnic differences in physical activity in children of the same age, that is ≥2000 counts per minute (CPM) for MVPA and <100 CPM for sedentary time. These cut-points are very close to those validated for children by Evenson et al. [24].

#### Physical activity interview

Each participant provided three 24-hour recall Physical Activity Interviews. Interviews took place during school hours on Monday, Tuesday and Wednesday, reporting on activities conducted on Sunday, Monday and Tuesday respectively. All Physical Activity Interviews were conducted on school grounds, in a private room, on a one-to-one basis. Each interview took between 10–20 minutes to complete. Interviews have been shown to have higher validity than self-administered questionnaires for the assessment of physical activity by self-report in children [25]. The 24-hour recall method reduces error caused by difficulties children may have remembering the duration of activities after 24 hours or more has passed [26]. The Physical Activity Interview is a previously validated tool [27], requiring the recall of all activities and the length of time spent in each activity on the previous day in a structured, chronologically ordered manner from waking to going to bed. Girls were prompted to recall chronologically all activities undertaken the previous day in the following time segments: before school, at school before lunch, at school after lunch, after school until supper and after supper until bedtime. As noted by Foley et al. [26] this method has the advantage that each activity sequentially cues recall of the following activity, and anchor points such as time of day, meals or school breaks are used to divide recall periods into manageable chunks. Participants estimated the duration of each activity. Girls were also provided with diaries and asked to prospectively note their activities during the day and these were used to prompt retrospective recall the following day.

For the purposes of this analysis we extracted quantitative data on the daily duration of specific behaviours of interest; participation in organised sport or exercise, outdoor play, screen-time, as well as information on mode of travel to school. Only activities reported outside normal school hours (and therefore ‘discretionary’), including participation in clubs at school after the compulsory school-day, were considered. Following the Health Survey for England [15], organised sports and exercise included time spent in organised sports, team sports or exercise activities such as dance or martial arts, whereas outdoor play included time spent riding a bike or scooter (other than for travel to school), kicking a ball around, skipping, playing on outdoor play equipment such as trampolines or playing chasing games. Time spent watching television, on a computer or playing on a games console was considered screen-time. Mode of travel to school was considered ‘active’ if a girl travelled on foot or by bicycle for at least one journey per day. Attendance at and mode of travel to mosque school were also recorded.

The distribution of variables assessing time spent in each of these behaviours was positively skewed. For this reason, and because of concerns about the accuracy of children’s assessment of the duration of activities, daily dichotomous variables were constructed for participation in sport and exercise (did or did not participate), outdoor play (did or did not play outdoors) and for screen-time (2 hours or less or more than 2 hours, based on current recommendations in the United States, Canada and Australia (but not the UK) that children should watch television or use other electronic media for no more than 2 hours per day [28-30]).

#### Day length and rainfall

Goodman et al. [31] found that longer day length was positively correlated with objectively measured physical activity levels in British primary school children, and that children played outdoors more on longer days. Day length was assessed as number of hours between 8 am and sunset. Rainfall is negatively associated with physical activity in British primary school children [31,32]. Information on rainfall on each day of data collection was obtained from a weather station that was situated between 2 and 5 miles from each school.

#### Parental interviews

Parental interviews were conducted in person and were semi-structured, guided by a list of topics related to physical activity and diet (dietary data not reported here). The physical activity topics centred on the participation of girls in organised sports and exercise, outdoor play, active travel and screen time, and parents were prompted to describe the family context for these activities. Most interviews were conducted in participants’ homes, but a number took place in private rooms in schools or community centres. All interviews were in English, with a Punjabi and Urdu speaking assistant present at interviews with British Pakistani parents where fluency in English had not been established prior to the interview. Interviews varied in duration from 35 minutes to 75 minutes. All interviews were recorded. YHT listened to recordings twice and transcribed all parts of the discussion that related to the girls’ physical activity levels and sedentary time or any associated behaviours or potential influences on these behaviours.

#### Statistical analysis

Statistical analyses were carried out using SPSS version 20. All accelerometer-based outcomes appeared normally distributed and tests for ethnic group differences were conducted using multilevel linear regression models. Tests for ethnic group differences in behaviours were conducted using multilevel generalised linear mixed models with a binary outcome. As the number of schools and children was relatively small, limiting higher level variation, a 2 level model was fitted (days nested within participants), with school included as 6 dummy variables. Models adjusted for school, year group, rainfall, daylight hours and weekend versus school day.

#### Qualitative analysis

Parent interview transcripts were read for information on participation in organised sport and exercise, outdoor play, use of screens, and mode of travel. Analysis was thematic and primarily inductive, based on the principles of grounded theory, in which theoretical insights emerge from the data rather than vice versa [33]. Following close reading of the interview transcripts, we identified key emerging themes, patterns and variation [34], which formed the basis of broad coding and analysis.

## Results

Of the 9 schools approached, 7 agreed to participate. In three schools the researchers approached only year 5 girls, because head teachers were concerned about the impact of participation in the study on upcoming public examinations for year 6 girls. Two hundred and fifty three girls were invited to participate in the study, of these 189 (75%) girls were recruited into the study, and 166 (88%) were British Pakistani or White British. One-hundred and forty-five (70 White British and 75 British Pakistani) girls provided at least 3 days of at least 500 minutes of accelerometer data, including 2 weekdays (and therefore 1 weekend day), and were included in analyses. Of these 145 girls, 132 (91%) provided data for 4 days.

There was no difference in age between the White British and British Pakistani girls (Table 1). British Pakistani girls were more likely than White British girls to come from households where parents were married or cohabiting and where there were 4 or more children in the household (reported for nearly half of British Pakistani families) and to have a mother who looked after the home rather than being in paid employment (Table 1).

**Table 1:** Sociodemographic characteristics of the girls in the final sample

	**White British**	**British Pakistani**
Number	70	75
Age (years)	9.9 ± 0.7	10.0 ± 0.7
School year group		
5	51 (73%)	53 (71%)
6	19 (27%)	22 (29%)
Parental marital status*		
Married/cohabiting	37 (53%)	53 (71%)
Single	33 (47%)	22 (29%)
Number of children in household***		
1	16 (23%)	3 (4%)
2 or 3	45 (64%)	38 (51%)
≥4	9 (13%)	34 (45%)
Mother’s employment status (N = 139)***		
Employed or student	37 (54%)	11 (16%)
Unemployed	15 (22%)	10 (14%)
Looking after the home	16 (24%)	50 (70%)
Mother’s country of birth (N = 139)		
UK	70 (100%)	32 (44%)
Pakistan	-	37 (56%)
Father’s country of birth (N = 132)		
UK	69 (100%)	27 (43%)
Pakistan	-	36 (57%)

*p ≤ 0.05 by chi-squared; ***p ≤ 0.001 by chi-squared.

### Accelerometry

**Figure 1:**
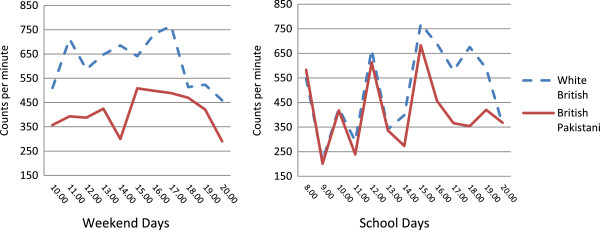
Average counts per minute by hour on weekend and school days.

There was no significant difference in average registered time for each group (Table 2) (overall adjusted means and 95% confidence intervals (CI) for British Pakistani girls 795 (776, 814) minutes and for White British girls 774 (756, 792) minutes). Mean counts per minute and minutes spent in MVPA per day were significantly lower for British Pakistani than for White British girls (overall adjusted means 417 CPM (381, 452) and 519 CPM (484, 553) and 52 minutes (47, 57) and 66 minutes (61, 71) respectively). British Pakistani girls spent significantly more time sedentary per day (Table 2) (542 minutes (531, 553) compared with 514 minutes (503, 525) for White British girls).Hour by hour activity counts per minute for weekend days and school days (Figure 1) show that White British girls were more active than British Pakistani girls throughout weekend days, and that activity levels diverged on school days from 3 pm, when school finished.

**Table 2:** Accelerometer derived variables averaged for weekend days and school days (raw values)

	**Weekend days**		**School days**		Corrected mean difference overall (95% CI)*
	**White British**	**British Pakistani**	**White British**	**British Pakistani**	
	**N = 75**	**N = 70**	**N = 75**	**N = 70**	
Registered time (minutes)	742 ± 97	749 ± 82	783 ± 81	829 ± 67	21 (−3, 45)
CPM	585 ± 239	411 ± 173	493 ± 133	398 ± 104	−102 (−145, −59)***
MVPA (minutes)	70 ± 29	46 ± 22	65 ± 19	54 ± 19	−14 (−20, −8)***
Sedentary time (minutes)	483 ± 88	523 ± 83	535 ± 68	583 ± 64	28 (14, 42)***

*Estimates for differences between White British and British Pakistani girls derived from multilevel models adjusted for school, year group, weekend/weekday, daylight hours and rainfall and, for sedentary time only, registered time.

### Physical activity recall interviews

There was a significant ethnic group difference in reported participation in organised sports and exercise and in outdoor play, with White British girls more likely to report each activity (Table 3). There was no significant ethnic difference in the proportion of girls reporting over 2 hours of screen-time per day in adjusted models (Table 3).

**Table 3:** Reported behaviours for White British and British Pakistani girls

	**Sunday**		**Monday**		**Tuesday**		**Odds ratio†** **(95% Confidence intervals)**
	**White British**	**British Pakistani**	**White British**	**British Pakistani**	**White British**	**British Pakistani**	
	**N = 68**	**N = 73**	**N = 64**	**N = 65**	**N = 67**	**N = 69**	
Participation in sport or organised exercise	7 (10%)	0	4 (6%)	3 (5%)	8 (12%)	6 (9%)	0.22 (0.08, 0.64)**
Outdoor play	50 (74%)	49 (67%)	38 (59%)	32 (49%)	46 (69%)	40 (58%)	0.42 (0.20, 0.91)*
Screen time (more than 2 hours per day)	39 (57%)	29 (40%)	24 (38%)	11 (17%)	20 (30%)	12 (17%)	0.88 (0.45, 1.73)
Active travel to school			52 (81%)	39 (60%)	60 (88%)	46 (67%)	0.26 (0.10, 0.71)**

*p ≤ 0.05, **p ≤ 0.01.

^†^Odds ratio derived from generalised linear mixed models for repeated measures (adjusted for school, year group, daylight hours and rainfall on each day). White British girls are the reference group.

Most girls of both ethnicities travelled actively to school each day, but a significantly higher proportion of British Pakistani than White British girls used a non-active mode of travel (in all cases car) (Table 2). Walking was the most common mode of active travel, with only 6 girls, all White British, reporting travelling by bicycle. No girls reported travel by public transport.

The majority (48 or 65%) of British Pakistani girls attended mosque school after school and of these 45 (94%) attended on both school days. The average reported start time for mosque school was 4.35 pm (±40 minutes) and the average finish time was 6.20 pm (±41 minutes). Of the girls who attended mosque school 26 (54%) reported walking both ways while the remainder travelled by car both ways.

### Parental interviews

Nineteen parental interviews were conducted: 9 with White British parents (6 mothers, 1 father, and 2 mother-father couples) and 10 with British Pakistani parents (8 mothers, and 2 mother-father couples). The parent interviews shed light on some of the possible reasons for similarities and differences in White British and British Pakistani girls’ engagement in active behaviours and in screen-time. As will become apparent in the following section, there is considerable diversity in the views and experiences of parents within each ethnic group, as well as, in some cases, between groups. In considering each kind of activity we pay particular attention to the contexts in which they are undertaken, and to constraints on, or facilitators of, participation.

### Sports and organised exercise

Most parents of both ethnicities were keen to express how active their daughters were. In some cases this was emphasised in the absence of participation in organised sports and exercise outside school, but a number of White British parents listed a variety of organised sports and exercise activities their daughters participated in during the week. These included, but were by no means limited to, clubs taking place in school at the end of the school day.

*Thursday she does two and a half hours of dancing. Friday she does ballet from 5-6 pm. Saturday she dances 12–2.30…Sunday 10–12 she goes to gym.* White British (WB) Mother.

*On Friday she dances. On Saturday swimming and dance, Sunday horse riding and dance.* WB Mother.

Some British Pakistani parents also reported that their daughters took part in regular sports and exercise, although commitment to frequent organised sport was less evident in this group and parents more often referred to sport and exercise undertaken as a family group than to formal organised sessions. These activities are discussed below as outdoor play.

Parents of both ethnicities reported clashes between other aspects of family life and their daughters’ participation in sport or exercise and it was clear that access to sports and exercise was negotiated in the context of other demands on time. In one White British family, the daughters’ dance commitments took precedence over other family activities and in one British Pakistani family children were allowed to miss mosque one day a week in order to participate in after-school clubs. In both these cases the desires of daughters to participate in organised sports or exercise were accommodated by parents, although clearly this was on a more limited basis for the British Pakistani family:

*In half term they have dancing competitions on, so we can’t go anywhere, can’t do anything, as they have no time off. That does annoy me like, but the kids like it so….* WB Father.

*Every term the school runs a club, gymnastics, drama, dance..... One day a week I let them have a day off from mosque as they need to do something.* British Pakistani (BP) Mother.

Sometimes, however, other commitments curtailed participation in organised sport:

*We used to take her to gymnastics, but we can’t take her, because we work and we struggled to get her there.* WB Mother-Father.

*It’s too hectic when they come back from school. They only have an hour or so before they go to mosque which is half five or whatever.* BP Mother.

Mixed-sex sports were mentioned as an issue by some British Pakistani parents, but they generally found it acceptable for girls to participate in mixed sex activities at this age. One mother made it clear that she was happy for her daughter to participate in sports and exercise sessions with boys, and to continue mixing with boys as she gets older:

*I don’t mind the activities with boys and girls as when they get bigger they’ll have to go to university together. And they have boy cousins the same age and they play with them.* BP Mother.

### Outdoor play

Several parents of both ethnicities referred to their children’s eagerness to play outside:

*Well, nine times out of ten they want to play out. If they’re bad [ill] that’s the only time they want to stay in.* WB Mother.

*Every night she’ll come home and she’s out playing, playing football, out on the scooter, on her bike, she’s always out, she always comes home sweating and red.* WB Mother.

*She comes back from mosque at 6.30, has her dinner, then she plays in the back, on the bike, roller-skates. Her cousins play out too, for one hour, then she comes back in.* BP Mother.

Some outdoor play took place with other members of the family, especially in parks, a resource utilised by families of both ethnicities:

*We like Albert Park. I’ve always took her, from when she was a baby, so we go there quite a lot.* WB Mother.

*If it’s summer time then the oldest daughters will take the other two girls to the park and spend an hour or two there.* BP Mother.

*I play cricket with the kids. I try to…spend as much time with them as I can, to encourage these sorts of things. …They come over to me and say “Dad, let’s go play football…”* BP Father

Parental concerns about traffic or about personal safety limited opportunities for unsupervised outdoor play for some children, particularly those living in city-centre terraced (row) housing without gardens:

*She plays out more at her Dad’s as round here that road can be quite busy, but round near her Dad’s is more quiet and she has a lot of friends there.* WB Mother.

*Obviously some parents let their kids play out, that’s their choice, I’m not knocking them for it, but we won’t let them do it…because of other people around here.* WB Father.

*Now I think you can’t let your kids out on the street, on the doorstep even. We don’t trust, you know, people.* BP Mother.

*I do worry, but when I see other peoples’ kids playing out I think it’s alright. I keep a check on them, have family around and about.* BP Father.

Some of these concerns were explicitly linked to gender, by both White British and British Pakistani parents. Girls were considered more vulnerable than boys.

*She says “Well I’m a girl, I can do just as much” as her brother. You’re scared though aren’t you? You’re scared to let them out around here.* WB Father.

*She does play out, but I don’t like her to wander off too far. The boy, he wanders off a few blocks.* BP Father.

For most families the perceived dangers of outdoor play came from the environment and strangers. However, one British Pakistani mother’s concern for girls was expressed specifically in relation to the possibility of her daughters behaving inappropriately when outdoors and away from supervision.

*I want them to be in an environment where there’s no temptation from boys or anything. Here they might say they are doing things like going to school or going to town and they could not be… Young girls aren’t allowed to do that stuff there [Pakistan].* BP Mother.

This position was clearly contested within the family however, as her husband interjected that temptations are found *“wherever you go”.* No such concerns were expressed by any of the White British families interviewed.

### Active travel

Many parents reported that their daughter walked to school, sometimes alone but more often with a friend or with parents. Specific reasons for not walking to school included the journey being difficult for younger siblings:

*We don’t walk, we have the car. The little one gets tired and wants carrying everywhere so it’s too much. It’s easier in the car.* BP Mother.

Some White British parents, but not British Pakistani parents, mentioned that their need to get to work in the mornings meant that they dropped their children off at school by car because they used their car to travel on to work. Often the need to get to both school and work in the morning meant that White British parents did not feel they had time to walk to school.

*I’d struggle if I didn’t have the car as I start at 8. I need to get the kids up and to breakfast club*^*a*^*. It’s always a mad rush.* WB Mother.

#### Screen-time

Some parents of both ethnicities considered that time spent in front of television, games consoles and computers represents a threat to children’s health, and were at pains to emphasize their efforts to limit screen time, some reporting that their efforts were successful, others that they found it difficult to keep children away from screens.

*We don’t watch a great deal of TV. Sundays are no TV days…* WB Father.

*She can watch TV or play on her DS for half an hour at bed-time.* WB Mother.

*They have the attitude that they only need to come off when someone tells them to come off. I tried to control it loads of times, where you cut it off at a certain time, but they end up nagging you and that and you forget.* BP Father.

However, some British Pakistani (but not White British) parents expressed appreciation of the fact that during frequent visits between members of their extended family personal screens kept their children occupied, or adults and children watched television together. For example, two parents reported children watching television or taking screens or games with them during family visits:

*If I’m at work they probably go round to her parents’ house, spend some time there…the women just tend to sit about doing their thing and the kids, they have the games consoles to occupy them.* BP Father.

*We don’t really make plans to go out. We have the family round and just tend to stay in and watch stuff on the telly.* BP Father.

## Discussion

We provide objective evidence that British Pakistani girls were less active than White British girls, both in terms of average activity, assessed by counts per minute, and in time spent in moderate to vigorous physical activity. In addition, British Pakistani girls spent significantly more time sedentary than White British girls. These results echo those of previous comparisons of British South Asian and White British children using accelerometry or pedometry [9-11], but are the first to show that British Pakistani girls, specifically, were less active and more sedentary than White British girls. There are no published studies of activity levels in children of South Asian origin living in other Western countries.

In line with studies of adults of South Asian and White European origin in the UK [35-37] and descriptive data for girls from the Health Survey for England [15] we found that British Pakistani girls were less likely to participate in organised sports and exercise than White British girls. While most parents of both ethnicities were keen to describe their daughters as active, demonstrating an awareness of the health benefits of physical activity, British Pakistani parents were less likely than White British parents to emphasise the participation of their daughters in organised sports and exercise. As in Bentley et al’s [38] study of British parents of young children, parents of both ethnicities mentioned that other activities and duties of both parents and children acted as barriers to participation in organised sports and exercise, although some families re-arranged other commitments to accommodate their daughters’ organised sports and exercise. Parental employment was mentioned as one such barrier, and may be more important for White British girls, whose mothers were more likely to work outside the home. For British Pakistani girls, attendance at mosque school on weekday evenings was mentioned as factor limiting participation in organised activities, as previously found by Pallan et al. [39].

Most girls of both ethnicities reported playing outdoors (other than at school) at some point during the three days. However, British Pakistani girls were significantly less likely to report outdoor play than White British girls. The interview results suggested that some parents of both ethnicities, particularly those living in the inner-city, were concerned about their daughters playing outside because of perceived danger from traffic and other people. These kinds of concerns are often reported by parents [38,40,41] and the specific worries expressed here about girls are consistent with previous work showing that perceptions of neighbourhood safety were associated with objectively measured physical activity in girls (but not boys) [42]. British Pakistani parents did not express obviously different or greater concerns about outdoor play from White British parents.

British Pakistani girls were less likely to travel actively to school than were White British girls. Owen et al. [16] also found that South Asian children in general were less likely than White European children to walk or cycle to school. Coping with children of different ages during the walk to school was mentioned as an inhibiting factor for British Pakistani families, who had more children per household than White British families. Immigrant Somali families in the United States reported similar problems related to having relatively large numbers of children and thus needing to take preschool children on the walk to school, and children of different ages to different schools [43]. Consistent with previous studies [44], parents of both ethnicities also reported that time constraints limited their ability to accompany children to school on foot and, as for outdoor play, the concerns parents expressed about neighbourhood safety may have inhibited unsupervised active travel to school [44].

We did not collect information on distance between home and school, which is known to be strongly correlated with mode of travel, with children living further from school more likely to travel by car [44]. However, Owen et al. [16] found that South Asian children in their study lived closer to school on average than White British children, so that distance from school did not explain the greater likelihood of South Asian children to travel to school by car in their study.

Interestingly, given that British Pakistani girls spent more time sedentary than White British girls, fewer British Pakistani girls than White British girls reported daily screen-time of over two hours. However, there was no statistically significant difference between the groups in adjusted models. Others have also reported no difference in self-reported screen-time in British South Asian and White children [19,20]. It appears, therefore, that British Pakistani girls accumulated more sedentary time in other behaviours. Our recall and interview data suggest that possible behaviours that might contribute to higher sedentary time in British Pakistani girls include time at mosque school and socialising with relatives in the home.

The most prominent explanation in the literature for low physical activity levels in girls and women of South Asian origin has been the importance of South Asian/Islamic ‘culture’ and religious proscriptions in limiting physical activity outside the home [45]. For example, Carrington et al. [46], based on interviews with 114 young South Asians (male and female, ages 11–24 years) in a northern English city, found that parental disapproval, particularly in relation to activities that could bring their daughters into contact with males, was a major barrier to sports participation, and that the need to uphold ‘*izzat*’ (family status and honour) played a key role in the willingness of adolescent girls/young women to participate in physical activities outside the home. As Kay [47] points out, those adhering to more “traditional” interpretations of Islamic teaching are likely to consider it important to conceal the female body from male view after puberty and thus to consider participation in sport inappropriate unless it takes place in a single-sex setting. Thus, the (non-) existence of single sex facilities for organised sports and exercise (alongside restrictive dress codes) is frequently mentioned in the literature as a potential barrier to physical activity for South Asian girls and women [45,47,48]. In general, as others have noted, there has been a tendency to homogenise the experience of Muslim girls and women and to reify ‘religious and cultural traditions’ that constitute barriers to physical activity [47,49-51].

However, such concerns were not emphasised by most British Pakistani parents in our study. There was relatively little evidence that participation in physical activity outside school in the form of mixed sex sports and exercise sessions, or in unsupervised outdoor play or walking, was actively prohibited for these young girls; one British Pakistani mother did express anxiety about ‘temptation from boys’, but this was not a widely-expressed worry among our study participants. It is certainly possible that family concerns about British Pakistani girls’ activities and social interactions may have influenced the activity levels of girls in our study (e.g. on the school playground [52]). However, for the young girls of both ethnicities in our study it appears to have been the more prosaic constraints such as time and managing other family commitments together with widely-shared fears about physical safety, that were a more immediate limitation on physical activity outside school. This may reflect the young age of our participants, but also highlights the existence of a wide range of possible constraints on girls’ activity. There was evidence of differences between the ethnic groups in the nature of family commitments outside the school day, and in structural constraints on activity, such as maternal employment and family size.

### Study limitations

It is important to acknowledge that our interview data were fairly limited. The parents of only 21 girls from among the 166 study participants volunteered to be interviewed, and just 19 interviews were carried out. Although this kind of qualitative research does not require a statistically representative sample, in that insights derived are intended to be illustrative rather than generalisable, the fact remains that this was a highly self-selected sample of parents, who may have held particular views about the value of physical activity and who were perhaps keen to share their positive experiences. In relation to the point about ‘cultural’ or ‘religious’ barriers to participation in sports and exercise, it may be that a wider range of opinions from parents might have suggested a rather different picture.

Our analyses do not adjust for socioeconomic status. Owen et al. [9] reported that adjustment for parental occupational social class did not change the differences in patterns of physical activity they reported between South Asian and European children, which were very similar to those we report here. However, it is possible that socioeconomic status affects the behaviours assessed here and future studies would benefit from appropriate assessment of socioeconomic status. For example, it is possible that British Pakistani families were more likely to live in inner-city housing without gardens than White British families and that this limits outdoor play in British Pakistani girls.

While screen-time is likely to be the most common sedentary behaviour among young girls [53], future studies should assess participation in other sedentary behaviours, such as time spent sitting and socialising with family and friends or at mosque school, that might help explain why British Pakistani girls were more sedentary than White European girls.

### Study strengths

The strengths of this study lie particularly in the combination of methods, allowing not only objective characterisation of activity levels and time spent sedentary, but an exploration of the behaviours and family context underlying these observations. Thus, unlike previous studies, we have been able to provide potential explanations of our accelerometry findings.

## Conclusions

British Pakistani girls were less physically active than White British girls and engaged to a lesser extent in key activities (organised sports and exercise, outdoor play and active travel) known to contribute to objectively measured physical activity levels in children outside school. British Pakistani girls were also more sedentary than White British girls, although there was no significant difference in the proportion of girls who watched screens for more than the widely recommended limit of two hours.

Detailed insight from interviews suggests that structural constraints of family life limited the participation of girls of both ethnicities in physical activity. There was some evidence to suggest that these constraints varied between the two groups, particularly in relation to maternal employment, number of children in the family and obligations to the wider family, and attendance at mosque school. Parental concerns about safety for children were an important factor affecting participation in physical activity for both groups, while concerns about modesty, often highlighted in the literature on physical activity in girls and women of South Asian origin, had limited impact for the young girls in this sample.

Interventions designed to address low levels of physical activity in young girls should recognise the importance of these structural constraints and concerns, and possible variation in constraints across ethnic groups, avoiding the temptation to focus on high profile ‘cultural differences’. For example, the targeting of opportunities for single-sex organised exercise or play to girls of South Asian origin is unlikely to be effective unless provided at a time and place that fits with the wider commitments of girls and their families.

## Endnote

^a^Breakfast club is a childcare service that runs on school grounds between the hours of 7 – 9 am. This service includes breakfast and play activities for children who attend.

## Competing interests

The authors declare that they have no competing interests.

## Authors’ contributions

All authors contributed to the design of the study. YHT collected the data and lead the analysis, assisted by TP and, for qualitative data analysis, by KH. All authors contributed to and approved the final manuscript.
